# Can Targeting Hypoxia-Mediated Acidification of the Bone Marrow Microenvironment Kill Myeloma Tumor Cells?

**DOI:** 10.3389/fonc.2021.703878

**Published:** 2021-07-19

**Authors:** Gilberto Gastelum, Mysore Veena, Kylee Lyons, Christopher Lamb, Nicole Jacobs, Alexandra Yamada, Alisher Baibussinov, Martin Sarafyan, Rebeka Shamis, Jeffry Kraut, Patrick Frost

**Affiliations:** ^1^ Department of Hematology/Oncology, University of California, Los Angeles, Los Angeles, CA, United States; ^2^ Department of Research, Greater Los Angeles Veterans Administration Healthcare System, Los Angeles, CA, United States

**Keywords:** pH balance, acid base regulation, bone marrow microenvironment, hypoxia and apoptosis, multiple myeloma

## Abstract

Multiple myeloma (MM) is an incurable cancer arising from malignant plasma cells that engraft in the bone marrow (BM). The physiology of these cancer cells within the BM microenvironment (TME) plays a critical role in MM development. These processes may be similar to what has been observed in the TME of other (non-hematological) solid tumors. It has been long reported that within the BM, vascular endothelial growth factor (VEGF), increased angiogenesis and microvessel density, and activation of hypoxia-induced transcription factors (HIF) are correlated with MM progression but despite a great deal of effort and some modest preclinical success the overall clinical efficacy of using anti-angiogenic and hypoxia-targeting strategies, has been limited. This review will explore the hypothesis that the TME of MM engrafted in the BM is distinctly different from non-hematological-derived solid tumors calling into question how effective these strategies may be against MM. We further identify other hypoxia-mediated effectors, such as hypoxia-mediated acidification of the TME, oxygen-dependent metabolic changes, and the generation of reactive oxygen species (ROS), that may prove to be more effective targets against MM.

## Introduction

The underlying factors resulting in the development and progression of solid tumors is complex and multivariable, but tumor genesis is likely first initiated by the accumulation of multiple genetic mutations (the Knudsen hypothesis) (1) that result in changes of tumor growth, altered tumor phenotypes, alterations in immunogenicity, increased metastatic potential, enhanced malignancy, and development of chemo- or immunoresistance. Tumor progression is further driven by selective pressure that develops within the tumor microenvironment (TME) in response to these changes that selects for more malignant phenotypes. As solid tumors typically outgrow the ability of the vasculature to provide sufficient nutrients and oxygen levels to support cell growth, factors like hypoxia-inducible transcriptional factor (HIF) ameliorate this by activating multiple rescue and salvage pathways (i.e. angiogenesis). Since these survival pathways underlie the ability of cancer cells to survive inimical low pO_2_ conditions within the TME, targeting these specific survival pathways (especially in solid tumors), either by genetic manipulation or pharmacological intervention of HIF is a promising and frequently proposed anti-cancer strategy. In an analogous manner to solid tumors, similar anti-tumor strategies have been proposed for hematological or blood-derived tumors such as MM. Despite numerous clinical studies and some modest efficacy in a wide array of cancer types, the anti-tumor effectiveness of these treatments has generally been limited and short-lived. In fact, some evidence suggests that hypoxic conditions in the TME may actually support more malignant cancer phenotypes. For example, since the bone marrow is already hypoxic and the relative low levels of O_2_ may actually support myeloma progression, this raises questions about how effective targeting HIF (or angiogenesis) in killing hematological tumors will be, especially in light that these cells may already be adapted to very low pO_2_ levels. In this review, we propose that targeting alternative pathways, such as hypoxia-mediated acidification of the TME, oxygen-dependent metabolic changes, or the generation of reactive oxygen species (ROS) may offer more effective clinical therapies as these events represent obligate cellular responses to low pO_2_ that must occur for the tumor cells to survive.

MM develops from malignant plasma cells that have migrated out of peripheral germinal centers and which have engrafted in the bone marrow (BM) (2). As the second most common hematological malignancy, MM accounts for 1-2% of all cancer related deaths (3). Each year approximately 20,000 new cases are diagnosed in the United States with older male patients having increased risk. African Americans are more than twice as likely to develop MM than Caucasians for reasons that are unclear. Despite some success in prolonging patient survival with new therapeutic strategies this disease remains incurable (4).

Myeloma tumor cells invade, grow and thrive within the BM, a complexly organized tissue characterized by heterogeneously distinct cellular and molecular “microniches” (5–8). These niches can be defined in many ways, but the BM regions characterized by low oxygen levels (hypoxia) are of special interest. In most solid, non-hematologically-derived tumors, cancer cells undergo an initial metastasis when they invade and engraft themselves at sites distant from their origin. Solid tumors can outgrow the ability of the vasculature to supply oxygen and nutrients, resulting in conditions of low pO_2_ that has been implicated in increased cancer cell malignancy and development of resistance to chemo- and radiation therapy (9, 10). Yet, whether or not BM engrafted MM malignancies develop along similar lines due to hypoxic conditions analogous to what is observed in non-hematologically-derived solid tumors is unclear. Since much of the rationale for targeting the hypoxic niche in MM has been based on studies in solid tumors, it is important to clarify how the MM niche may be different. For example, while the exact relationships between MM engrafted tumor cells and the hypoxic BM microniches remains mostly undefined, evidence suggests that the innate low pO_2_ within the BM actually provides a critical protective advantage to MM tumor cells (8, 11, 12). In fact, the innate low pO_2_ in the BM TME has been linked to the promotion of “stemness” in MM tumor cells (13) and this may represent a much different TME-environment that develops in solid tumors following engraftment of metastatic cancer cells migrating from a distant primary tumor. In the former situation, MM cells may already be adapted to low pO_2_ and be less reliant on transcriptional activation of *de novo* angiogenic pathways (say as mediated by VEGF), than in solid tumors.

In solid tumors, low pO_2_ levels in the tumor nodule results in the subsequent activation of a suite of hypoxia-inducible transcriptional factors (HIFs) that are important for upregulating about 100-200 genes responsible for mediating the adaptive hypoxic response of these tumor cells. Presumptively, these genes activate salvage pathways, including pro-angiogenic signals and altered cancer cell metabolic activity (the Warburg effect) that are important for the cancer cells to survive the increased oxygen burden within the TME (9). While most non-cancer cells are generally unable to survive in these low oxygen conditions and undergo hypoxia-mediated apoptosis, some tumor cells seem to “thrive” in low pO_2_ conditions, implying that hypoxia, in and of itself, is not necessarily sufficient to kill cancer cells or that the cellular adaptive response to low O_2_ selects for more malignant phenotypes. In fact, a growing consensus suggests that hypoxia is strongly correlated with increased tumor cell malignancy (14), increased metastatic potential (15) and increased cellular resistance to chemo- and radiation therapy (16).

Since HIF is likely a critical “master-regulator” of the cellular response to hypoxia, it is suggested that inhibiting the ability of HIF to induce these adaptive hypoxic responses makes it an attractive anti-cancer strategy for killing both solid and hematological tumors, like MM (11, 17–19). Treatment of MM with a hypoxia-activated pro-drug (20), the suppression of HIF (with small inhibitory RNA) (21), or a specific HIF-targeting polyamide (11) all showed some anti-MM effects *in vitro* and *in vivo* and some of these strategies have moved to clinical trials (22). Despite the wide range of novel therapeutic strategies designed to target hypoxia and HIF-mediated salvage pathways (i.e. anti-VEGF/angiogenesis), the clinical effectiveness are relatively modest (23, 24). This raises the question of whether or not targeting HIF (or even down stream hypoxia-mediated angiogenesis) to further induce lower O_2_ levels (for example by blocking *de novo* neovascularization of the tumor nodule) is a therapeutic dead end as an effective strategy to treat hematological malignancies.

On the other hand, development of hypoxic TMEs are a common motif of both solid and hematological tumors which raise the question if better or more effective alternative downstream responses triggered by low pO_2_ can be developed, away from angiogenesis. In this case identifying obligate cellular response to low pO_2_ that must occur in response to hypoxia would be attractive therapeutic targets. For example, cellular hypoxia results in a marked decrease in the intracellular pH (pHi ~6.7) of cancer as a result of the tumor cells switching to anaerobic glycolysis and the concomitant production of lactic acid, a process that has been known for decades to occur in solid tumors (25). Under these conditions, cells must maintain their internal pH (pHi) at obligate neutral/basic levels (pH ~7.2-7.6) by removing or altering the acidic byproducts to the external environment (26, 27). The failure to maintain internal pH at obligate neutral/basic levels will result in the pH-mediated inactivation of enzymatic function thereby resulting in cell death. Thus, it is reasonable to argue that a critical aspect of the adaptive hypoxia-mediated response must also include the activation of rescue pathways responsible for maintaining the pHi at prescribed levels in both solid and hematological tumors. Simultaneously, the regulation of the internal pH will result in an acidification (~6.4-6.7) of the extracellular pH (pH_e_), as protons are pumped out of the cell, and that the acidification of the pH_e_ may impact on the TME in other ways, yet to be discovered. Since these are apparently obligate events that must occur in cells faced with low pO_2_ levels, regulation of the internal pH and acidification of the external pH, we propose that targeting hypoxia-mediated acid/base regulatory survival pathways may prove to be more effective anti-cancer therapeutic targets.

## Hypoxia in Solid Tumors

The six most common types of solid tumors (breast, lung, prostate, colorectal, melanoma and bladder cancers) account for about 1 million new cases per year. In most cases a primary mass within the tissue of origin develops and is frequently followed by individual cancer cells that break free and metastasize to distant regions of the body (including the bone). Despite the complex sequelae underlying tumorgenesis and metastasis, once established (either as a primary or secondary metastatic tumor), the growing cancer nodule typically outgrows the ability of the local tissue vasculature to provide sufficient levels of O_2_ and nutrients. This process results in the development of temporally and spatially diverse microniches in the tumor that are characterized by (1) “normal” pO_2_ levels, (2) areas of “low/hypoxic” pO_2_, and (3) necrotic regions. As such the response of cancer cells residing within these heterogeneous microniches will also be diverse. Normal, non-cancer cells and less malignant cancer cells are more likely to succumb to these inimical hypoxic conditions, whilst more malignant and resistant phenotypes will be selected. While the role of O_2_ and metabolism is well studied in solid tumors, it is less clear if the same conditions and pathways exist in hematological tumors. We postulate that while there are many similarities, significant differences do exist (**Table 1**) and such should be taken into consideration when trying to develop novel strategies to target these types of tumors. Yet an important question remains to be answered is if the cellular response to low pO_2_ (either in part or in toto) represent a critical survival pathway that must be initiated by cancer cells (and as such can be targeted) or if this is simply an epiphenomena linked to increased oxygen burden that the tumor cells have already bypassed.

**Table 1:** Summary table comparing oxygen-related similarities and differences between hematological and solid tumors.

	Hematological malignancies	Solid tumors
Major Types	Leukemia (develops in the bone marrow and travels through the bloodstream affecting blood cells) Lymphoma (develops and affects cells in the lymphatic system including lymphocytes and lymph nodes) Myeloma (develops in the bone marrow and affects plasma cells)	Sarcomas (tumors in a blood vessel, lymph vessel, ligament, bone, muscle, tendon or fat tissue) Carcinomas (tumors in epithelial cells; skin, glands and linings of organs like bladder and kidneys)
Response to Hypoxia	It was initially thought that hematological malignancies acted the same as solid tumors, but after targeting VEGF and angiogenesis with moderate success, research has switched to more modern approaches including targeting pH and metabolism	Neovascularization (angiogenesis) *via* release of hypoxia-inducible angiogenic factors like VEGF is crucial for the survival of solid tumors. Targeting angiogenesis and VEGF can be effective against solid tumors: Anti-VEGF drugs like bevacizumab (Avastin) and Sunitinib and anti-angiopoietin pathway agents like AMG 386 and Regorafenib are shown to be effective in various solid tumor cancers (like breast cancer, renal cell carcinoma, ovarian cancer, colorectal carcinoma, etc.)
	For MM, this is probably due to the already hypoxic environment of the BM paired with the large blood vessels present, reducing the viability of angiogenesis and micro vessel density playing a role in tumorigenesis.	
Physiological	Hematological malignancies do not show clear oxygen-dependent regions. Instead, clonal cells get dispersed unevenly based on anatomy (more clones at extramedullary sites).	Solid tumors have distinct and spatially heterogenous regions. There are three types of tissue regions based on oxygen distribution: Normoxic (well-oxygenated), Hypoxic (oxygen-insufficient) and Necrotic/Anoxic (oxygen- depleted).
	The malignant plasma cells here grow independent of another and thus do not form a solid nodule.	Malignant cells grow in conjunction to each other, forming a solid mass.
Cellular	Hematological malignancies adapt by inducing various gene expression patterns, including stabilization of HIF. However, the success of targeting HIF in these malignancies has not been especially effective (as noted for solid tumors).	Solid tumor cancer cells can adapt to changes in the microenvironment by inducing expression of various genes. HIF-1 is one such master regulator of the adaptive cellular hypoxic response.
Metabolism	In MM and other hematological malignancies, glycolysis is adjusted in a series of ways: Hexokinase II(HKII) is overexpressed, 3-bromopyruvate (3BP) has alkylation properties and can inhibit HKII leading to reduced ATP production and viability, PKM2 (an isoform form of Pyruvate Kinase) is upregulated, Acetyl-CoA is increased, lactate is present, all resulting in acidosis.	Glucose is fundamental for a cell’s metabolism as it leads to glycolysis which leads to the tricarboxylic acid cycle (TCA) and then oxidative phosphorylation allows for the production of ATP. Tumorigenesis relies greatly on the shift in glucose metabolism: from oxidative phosphorylation back to glycolysis even in a state of sufficient oxygen. Glycolysis is an inefficient metabolic pathway for normal cells, but has advantages for tumor cells.
	Glutaminolysis appears to be enhanced in MM cells, related to the idea that MM cells are glutamine addicted. This appears to be related to the hypoxic environment of the BM. The MYC oncogene is upregulated in MM, which also enhances expression of glutamine transporters and represses inhibitors of Glutaminolysis.	Glutamine is critical for cells to function, as it enhances proliferation, differentiation, cytokine production and apoptosis. Glutaminolysis is the glutamine equivalent to glycolysis, as they both result in energy production and nucleotide synthesis. Glutaminolysis specifically results in amino acid synthesis and fatty acid synthesis.
	“Reverse Warburg effect” describes MM cells and their supply or lactate from the surrounding environment.	Together, Glycolysis and Glutaminolysis mechanisms enable the growth and survival of the solid cancer cell.
		Another thing to note is the Warburg effect, which is essentially an enhanced aerobic glycolysis due to the hypoxic environment of tumor cells, but it can occur under normoxic conditions too.

## Hypoxia in Hematological Tumors

### Bone Marrow

The BM environment is hypoxic by its nature and this is critical for the activity of hematological stem cells and may also be an important underlying cause of metastasis of various tumor types to the BM (28–30). This is an important consideration, as the BM is already hypoxic and it remains unclear if engrafted MM tumor cells establish a greater hypoxic gradient similar to what is observed in solid tumors. Hypoxia is described as an insufficient amount of metabolic oxygen resulting from a discrepancy between homeostatic oxygen supply and consumption. If the level of oxygen tension is below the physiologic level, then the tissue is hypoxic. Physiologic pO_2_ in “normal” tissues can vary based on the tissue vasculature and cellular metabolic needs, which is organ specific and can vary from 40-85 mmHg (31). Measuring the pO_2_ level is difficult and invasive, but in animal studies, the pO_2_ of the BM was found to range from 10-30 mmHg, which is hypoxic relative to most other tissues (32). While the microenvironment of BM is hypoxic in healthy individuals, the niche of engrafted MM can be even more hypoxic, but it is not clear if what drives this process and how much more hypoxic the microniche may become (20). In cultured MM cell lines, the cells can survive relatively low pO_2_, (down to 0.2%) without significant cell death (11). In fact there do not appear to be clearly defined and physiologically critical level of O_2_ (other than totally anoxic conditions) that represents a “lower” target limit of inhibited cell survival *in vitro* or *in vivo*. Thus, one aspect that is lacking in almost all studies is a demonstration of the *in situ* physiological effectiveness of anti-HIF or anti-angiogenic therapies must achieve a specific level of sustained tumor hypoxia in order to be toxic to the cancer cells. In fact, most tumor cells appear to quickly adapt to low pO_2_ levels in order to survive.

In contrast to solid tumors, hematological tumors derive from blood cells and are already associated with the microenvironments of the blood and lymphatic tissues, including hematopoietic stem cells living in the BM. The development of hypoxic conditions within hematological cancers and associated tumor masses (such as seen in lymphomas and myelomas) likely share some similarities with solid tumors but it is equally likely that they differ in many other aspects (**Table 1**) and that the low pO_2_ in BM may not strictly represent an inimical environment for hematological tumors. Leukemias, for example, do not form solid masses but rather are “liquid tumors” that circulate with the blood and lymphatic systems. In this case, the role of oxygen in these diseases was presumed to be inconsequential due to the environment of the blood, and has long been overlooked (33). Leukemias develop from the uncontrolled hematopoiesis of stem cells in the BM and it seems that hypoxia plays a critical role in maintaining these cancerous stem cells (34). In contrast, MM and lymphomas form “solid” tumor masses and likely share some physiological similarities to solid tumors, but again, the exact role of hypoxia in MM remains unclear (8, 30).

The development of MM is complex and not fully understood, but appears to be caused by genetic and chromosome abnormalities caused by genetic mutations during terminal differentiation of B-lymphocytes into plasma cells. In about half of all cases of MM, chromosomal translocations (primarily 11q13 (cyclin D1), 6p21 (cyclin D3), 4p16 (FGFR3 and MMSET), and 16q23 (c-maf) onto the immunoglobulin heavy chain of chromosome 14. In the other half, trisomies of odd-numbered chromosomes are frequently observed (35–37). Under these conditions of deregulated cellular proliferation, additional mutations can accrue, such as mutations in the RAS family of oncogenes (including K-ras and N-ras) that induce constitutive ERK activation and which are observed in about 20% of all MM cases (38). Interestingly, tumor hypoxia may actually facilitate genetic instability and as such may underlie the development of genetic lesions (39, 40).

MM progresses through stages, including monoclonal gammopathy of undetermined significance (MGUS) (with a 10% chance of progressing to MM), smoldering multiple myeloma (SMM) and multiple myeloma (MM). Individuals with MGUS are often asymptomatic with pre-malignant tumors made up of about 15% lymphoid or lymphoplasmacytoid MGUS and 85% plasma cell MGUS (41). Patients with symptomatic MM disease are characterized by skeletal destruction, anemia and renal failure. The initial stages of MM (such as MGUS) occur when long-lived plasma cells (PC) from peripheral lymphoid tissues migrate to the BM. While the exact mechanisms regulating this migration is unknown, some evidence supports the hypothesis that hypoxia is an important factor for the initial growth, survival, and proliferation MGUS cells migrating into the BM (42). Another theory is that this migration is mediated by initial hypoxia-induced induction of the epithelial-mesenchymal transition (EMT) processes (43). Other factors may also play a role in MM engraftment in the bone marrow, including: SH3GL3 mediated by the FAK/PI3K signaling pathway (44), Wingless/int (Wnt) family including the Wnt/RhoA pathway and protein kinase c (PKC) (45), CD40 activation which activates downstream AKT, phosphatidylinositol-3-kinase (PI3K), and nuclear factor (NF)-κB which targets urokinase plasminogen activator (uPA) (46), and SRC3 which regulates Cx43 expression (47, 48). Altogether, these findings suggest that MM may not actually alter the “normal” BM niche to make it more hypoxic as opposed to hypoxia and HIF activation representing epiphenomena of MM engraftment

### Angiogenesis and VEGF

For many years, a hallmark of MM progression and prognostic potential was the observed correlations to increased expression of angiogenic factors and increased microvessel density (MVD) in the BM of MM patients (49–52). Angiogenesis is necessary for essential biological processes such as embryogenesis (53) and wound healing (54), but is also necessary for tumor growth and may induce a dormant, benign tumor into a growing, malignant mass (55). The term “angiogenic switch” refers to a time-restricted event where pro-angiogenic factors outweigh anti-angiogenic factors resulting in advance tumor progression (56). The molecular mechanisms underlying a MM-induced angiogenic switch have been studied extensively, with a relatively large number of biological factors identified, including VEGF, bFGF, HGF, OPN, Ang-1, Ang-2, IL-6 and IL-8 (57). Despite this, it still remains unclear how important a role *de novo* angiogenesis and increased MVD plays in MM engrafted in the BM or whether this could simply be an epiphenomena associated with tumor-mediated changes in BM architecture. On the other hand, the drug thalidomide has demonstrated some efficacy against MM and has been linked to its possible anti-angiogenic properties (58).

### HIF and the Adaptive Cellular Hypoxic Response

The growth of *de novo* vasculature within a solid tumor typically does not develop like normal, healthy vasculature, but instead appears as disorganized, interconnected, leaky tubular structures (59). A combination of this flawed vascular system, the unsynchronized growth rates of tumor and endothelial cells, sluggish blood flow, and high interstitial fluid pressure contributes to highly variable and heterogeneous hypoxic environments within the tumor nodules (which includes fluid geographical regions of “normoxia”, regions of variable pO_2_, and areas of anoxia and necrosis). In response to the reduced tumor pO_2_ levels, a concomitant upregulation of multiple pro-angiogenic factors leads to increased tumor *de novo* angiogenesis acting as a salvage pathway, and as such, this has been seen a potential target for anti-tumor therapies attacking the cellular response to hypoxia.

The master regulator of this cellular hypoxic adaptive response is mediated, at least in part by HIF. The HIF transcription factor consists of constitutively expressed β-subunit (HIFβ) and inducible α-subunits (e.g. HIF1, 2, or 3α). HIF1α and HIF2α are frequently upregulated in MM patients (60). The upstream O_2_-dependent regulation is mediated, by prolyl-4-hydroxylase domain protein (PHD1-3) hydroxylation on proline residues of the α-subunits that target them for proteasome degradation. PHD activity is suppressed by low pO_2_, which increases HIFα stability, allowing for dimerization to HIFβ, and nuclear localization of the transcription factor to hypoxic response elements (HREs). However, PHDs control more than just HIF stabilization, with PHD3 emerging as a presumptive tumor suppressor gene (61) whose expression varies between different cell types and oxygen concentrations. Shah et al. (62) and Hatzimicheal et al. (63) reported frequent aberrant CpG methylation of PHD3 (but not PHD2) in MM clinical patient samples (42% and 33%, respectively). Furthermore PHD3 is downregulated in hypoxia-resistant MM cell lines and restoring its expression rescued O_2_-dependent regulation of HIF2α as well as sensitivity to hypoxia-mediated apoptosis (19). The recognition of HIF as a master regulator of the hypoxic cellular response has led to multiple and diverse pharmacological strategies to target this pathway in multiple cancer types (64), but like many other transcription factors, the “drugability” of anti-HIF compounds remains a real barrier. HIF regulates a relatively modest 100-200 genes in response to hypoxia, which may make identifying more effective HIF-mediated obligate cellular survival targets more attractive and feasible. In **Table 2**, we present a comparison of selected gene expression from the six most common types of solid cancers (breast, lung, prostate, colorectal, melanoma and bladder) compared to MM hematological cancers.

**Table 2:** Summary table comparing HIF/hypoxia gene expression in various solid and hematological tumors.

Gene	Breast	Lung	Prostate	Colorectal	Bladder	Renal	Multiple myeloma
HIF	Increased expression is associated with worse prognosis (65)	May play a role in maintaining tumor cell growth and metastasis (66)	Like in breast cancer, HIF expression is associated with worse prognosis (67)	May play a role in maintaining tumor cell growth (68)	Moderate correlation with HIF (69)	Frequently seen cancer type with VHL syndrome (70).	Reported to be elevated in MM samples and correlates with poor prognosis (50)
VHL	Not noted as being critical in breast cancer	Not noted as being critical in lung cancer	Not noted as being critical in prostate cancer	Not noted as being critical in colorectal cancer	Not noted as being critical in bladder cancer	Frequently seen cancer type with VHL syndrome (70).	Not noted as being critical in MM progression.
VEGF	Frequently elevated and associated with worse prognosis	Frequently elevated and associated with worse prognosis	Frequently elevated and associated with worse prognosis	Frequently elevated and associated with worse prognosis	Frequently elevated and associated with worse prognosis	Frequently elevated and associated with worse prognosis	Reported to be elevated in MM samples and correlates with poor prognosis (18, 50)
PHD 1-3	Down regulated and correlated to worse prognosis (71)	May be a potential prognostic factor for lung cancer (72)	May be a potential prognostic factor for prostate cancer (73)	Frequently down regulated and correlated to worse prognosis (74),	Not noted as being critical in bladder cancer	Associated with changes in VHL syndrome (70).	Frequently silenced in MM (19, 63)
CAIX	Associated with poor prognosis and disease progress (75)	Correlated with increased risk of relapse (76)	Associated with poor prognosis and disease progress (75)	Not noted as being critical in colorectal cancer	Associated with poor prognosis and disease progress (77)	Associated with changes in VHL syndrome (70).	Upregulated in MM cell lines. Inhibition of CAIX activity sensitizes MM cells to hypoxia mediated apoptosis (12, 19)

Most solid cancers will develop regions of hypoxia and will likely exhibit increased HIF and related gene products. It remains unclear if this is an expected outcome of “normal” tumor progress or is related to a genetic lesion associated with activation of an oncogene or inactivation of a tumor suppressor gene. In general, the prognosis and progression of most cancers directly correlate with altered hypoxia-related gene expression when compared to non-malignant cells.

### Regulation of HIF Transcriptional Factors in Cancer

In general, HIFα-subunits are not typically muted in cancer; however, six HIF1A small nuclear polymorphisms (SNPs) have been associated with breast, lung, colorectal (CRC), gastric, prostate, oral cancer, and renal cell carcinoma (RCC) (78). Upstream of HIF, the PHD3 gene is frequently silenced by CpG methylation of the PHD3 promoter and downregulation of PHD3 expression is frequently observed in glioblastoma (79, 80), colorectal cancer (74), soft tissue sarcomas (81) and breast cancer (71). Perhaps the most significant factor in dysregulation of HIF signaling in some cancer cells are related to inactivation of the von Hippel-Lindau (VHL) factor, which is responsible for recognizing hydroxylated α-subunits and targeting them for ubiquination and degradation. VHL is often inactivated in renal cell carcinoma (RCC) (70). Unlike mutations of HIF, Von Hippel-Lindau (VHL) syndrome is an autosomal-dominant, multi-organ, familial neoplastic syndrome that predisposes affected individuals to the development of benign and malignant tumors, The most common VHL-associated tumors are hemangioblastomas involving brain, spinal cord, and retina; RCC; pheochromocytomas and paragangliomas; and pancreatic neuroendocrine tumors (PNETs) (82). These “canonical” O_2_- dependent hypoxia regulatory systems can be bypassed or disrupted by gain of oncogene function or loss-of-function mutations in tumor-suppressor genes [i.e. phosphatase and tensin homolog (PTEN)], or epigenetic silencing of the O_2_-sensing pathways resulting in constitutive O_2_-independent stabilization and expression of the HIFα-subunits which may support tumor growth and survival (83).

### Downstream Targets of HIF 

Perhaps the most studied and frequently targeted hypoxia related target has been VEGF and other related pro-angiogenic growth factors (as discussed in **Table 2**). There have been over 80 types of drugs developed through preclinical studies and phase I-III clinical trials, with targeting of the VEGF axis, such as the blockade of VEGF receptors (VEGFRs) or ligands with neutralizing antibodies, and the inhibition of receptor tyrosine kinase (RTK) enzymes being the most common therapeutic strategies [for review see (84)]. Yet despite some initial success, the clinical efficacy of these treatments has been modest. Thus, it may be more effective to identify targets against the adaptive hypoxic response that would be more effective in the clinic.

## Acid/Base Regulation

While O_2_ is critical for life, it is also clear that under hypoxic conditions, tumor cell metabolism must shifts towards anaerobic glycolysis to keep up with cellular energy demands. This likely represents an obligate metabolic pathway that can be targeted against cancer cells. Lactic acid, the metabolic end product of anaerobic glycolysis, accumulates along with CO_2_, leading to acidification of the extracellular environment. In subcutaneous xenograft model of solid tumors, the average pH inside MM tumors was ~6.9 compared to pH of 7.4 outside of the tumors (85). It is the buildup of these acidic byproducts that necessitates activation of various acid/base regulatory salvage pathways within the cells, else the enzymes will be inactivated. For example, carbonic anhydrase IX (CAIX) plays a crucial role in maintaining intracellular pH within a neutral/alkaline range under hypoxic conditions (86). It was recently reported that the sera of patients with relapsed/progressed MM reacted with antibodies to CAI, II, IX and XII and patients with elevated CA autoantibody titers had a significant survival benefit over those who did not (87). Importantly, inhibition of CAIX leads to the inhibition of growth of primary tumors and metastases and depletes cancer stem cell populations in a number of preclinical studies and against various hypoxic tumors (88, 89). We have shown that MM cells treated with acetazolamide, a CAIX inhibitor, can be sensitized to hypoxia-mediated cell death (12, 19).

In addition to the CA isozymes, the Na^+^/H^+^ exchanger-1 (NHE1) is a ubiquitously expressed acid-extruding membrane transport protein, and upregulation is commonly correlated with tumor malignancy (90). Recently, the NHE1 inhibitor, amiloride, was shown to induce apoptosis in a broad panel of MM cell lines and in a xenograft model, supporting a role for targeting pH regulation as an anti-MM therapy (91). We also showed that targeting CAIX activity with amiloride could potentiate hypoxia-mediated cell death of MM cell lines in a HIF-dependent manner (12, 19). Thus, targeting the obligate requirements for MM cells to maintain their intracellular pH in a very narrow defined range may offer attractive new anti-cancer strategies.

### Metabolism

MM cells also undergo multiple changes in metabolism reflecting changes as the progress from MGUS to MM that can reflect changes in pO_2_ burdens. Using mass spectrometry, large differences were found in amino acid, lipid, and energy metabolism profiles when comparing healthy patients and MM patients. These differences were found between the profiles of plasma metabolites of MM and MGUS patients, demonstrating that metabolic pathways change during disease progression (92). Consequently, the presence of certain metabolites in the blood or BM could be used clinically as a way to detect progression from the MM precursors (MGUS or SMM) to MM as well as measuring responses to changes in hypoxia. One study examined the presence of 2-hydroxyglutarate (2-HG) for this purpose. 2-hydroxyglutarate is an oncometabolite already known to cause tumorgenesis in other cancers. This study found significantly higher 2-HG levels in the BM of patients with MM compared with those of MGUS, and found that in SMM patients, 2-HG levels were associated with a higher risk of progression to MM (93). Metabolic changes have also been shown to be associated with drug resistance. Comparison of metabolic profiles of melphalan resistant and sensitive cell lines showed a switch to aerobic glycolysis, in line with the Warburg effect seen in other cancers (94). In addition, melphalan resistance is associated with increases in the oxidative stress response and survival and proliferation signaling (94). Metabolic changes were also observed in MM cells resistant to proteasome inhibitors (bortezomib and carfilzomib). Cell lines resistant to these drugs upregulate proteins involved in metabolic regulation, redox homeostasis, and protein folding and destruction. Based on these findings, a potential mechanism for resistance could be metabolic adaptations that favor the generation of reducing equivalents such as NADPH (95).

## Reactive Oxygen Species and Metabolism

In studies of metabolism, the production of reactive oxygen species (ROS) paradoxically creates both a threat to and growth signals for MM cells. ROS are produced as byproducts of metabolism resulting from oxidative respiration, but other metabolic processes such as oxidative protein folding also produce ROS as by-products. These molecules all have properties that make them highly reactive with other biological molecules, so the overproduction of ROS is toxic to the MM cell (96). Reactive oxygen species are produced in large amounts by cancer cells due to their increased/altered metabolic activity and oncogene activation (97). This is particularly important in MM, since healthy plasma cells already have relatively high levels of ROS production as a result of their role as antibody producing factories (98). As a result, MM cells produce large amounts of ROS that need to be dealt with in order to prevent cell death. This is achieved through an oxidative stress response. The oxidative stress response includes multiple mechanisms to reduce the amount of ROS in the cell, such as the expression of antioxidant enzymes, which eliminate ROS. Therefore, the high rate of ROS production must be countered by an increased rate of antioxidant production (99).

Paradoxically, ROS also promote tumor growth by acting as signals for proliferation. For example, the release of H_2_O_2_ from mitochondria activates transcription factor NF-κB, which is activated by myeloma cells in order to increase proliferation and survival (100). ROS from the mitochondria may also activate the PI3K and MAPK pathways, which also promote tumorgenesis (101). Therefore, ROS can serve both a beneficial and antagonistic role in MM tumor growth, and cells must balance their ROS levels to maximize signaling while preventing toxic effects in a manner analogous to hypoxia.

## Summary

The natural growth and development of tumors (either solid tumors or hematological tumors) influences the tumor microenvironment as the cancer cells outgrow the available blood supply and establish areas of low pO_2_. In order to survive these potential inimical conditions, it is clear that the tumor cells must activate hypoxia-mediated salvage pathways, that include increasing angiogenesis and MVD and switching metabolic pathways from oxidative-phosphorylation to anaerobic glycolysis. Because these events are tumor specific and do not generally occur in normal cells, these cellular hypoxic adaptive responses offer tantalizing targets for cancer specific treatments. On the other hand, the hypoxia-mediated activation of salvage pathways also selects for more resistant tumor cell phenotypes, enhance the malignancy and metastatic potential these cells, and contributes to the development of resistance to chemotherapy and radiation treatment. Furthermore, the redundancy in hypoxia-sensing pathways and the establishment of a diverse, fluid, and heterogeneous microenvironment confounds and limits the effectiveness of any specific therapy targeting one particular niche at the expense of another. Altogether, this could explain the overall modest impact of targeting hypoxia and hypoxia-mediated pathways in cancer.

In solid tumors, the progression of growth in the tumor nodule results in a well characterized development of decreased pO2. Because of the critical role of oxygen in cell survival, it seems attractive to attack the pathways that regulate the cellular response to low pO_2_ *in vivo*. In contrast to solid tumor development, we believe that hematological malignancies, such as MM, have similar but unique responses to the innate low pO2 TME that already characterizes the BM. In fact, hypoxia in the BM may actually be critical events underlying the initial homing and engraftment of MM tumor cells. We propose that the critical aspects of MM that are engrafted in the BM is distinct from that seen in non-hematological-derived solid tumors. Because low oxygen levels are already part of the normal BM niche, targeting HIF, for example, may have limited anti-tumor effectiveness in treating MM. This observation is supported by multiple clinical studies demonstrating that targeting hypoxia or angiogenesis is only moderately effective. Building on this, we further believe that there are other obligate cellular responses to low pO2, such as acid, such hypoxia-mediated acidification of the TME, oxygen-dependent metabolic changes in MM cells and the subsequent generation of reactive oxygen species (ROS), that may prove to be more effective targets against MM.

These pathways seem to be less well-defined (and less targeted) pathways that tumor cells are obligated to remediate if they are to survive hypoxic conditions. For example, the Warburg effect results in the generation of acidic intracellular milieu that require activation of additional pathways necessary to maintain the cellular cytoplasm at neutral/basic levels. Thus, the increased proton transport in highly malignant cells not only allows them to survive in a hostile environment, but also facilitates their metastasis to other sites in the BM. Acid secretion by the cells may also affect the formation of osteolytic bone lesions through the modulation of osteoclast and osteoblast activity and that of acid-dependent proteases including cathepsins, serine proteases, and matrix metalloproteinases (MMP) secreted by the cells (102). Given the important role attributed to acidification of the TME and the regulation by HIF of the hypoxic adaptive response in generation and spread of the MM tumors, it is logical to target these factors for treatment of MM.

In summary, we propose that whilst targeting the cellular response to hypoxia in MM is attractive, we also believe that next generation anti-MM strategies must move beyond HIF and VEGF/angiogenesis. Further, we believe that the main focus should be in identifying those salvage or rescue pathways that MM tumor cells are obligated to activate in response to hypoxia, such as the build of acidic byproducts are result of metabolic switching to aerobic glycolytic pathways when the pO_2_ is low. These strategies could be developed alone, or in combination with other HIF/hypoxia targeting modalities in order to synergize the anti-tumor effects in the clinic.

## Data Availability Statement

The original contributions presented in the study are included in the article/supplementary files. Further inquiries can be directed to the corresponding author.

## Author Contributions

PF developed the research, JK made significant contributions to the direction of the research. VM, GG, KL, AY, CL, MS, NJ, and AB equally performed experiments and collected/analyzed data. KL and CL contributed equally to the writing of this work, RS significantly assisted in writing and preparing the manuscript. All authors contributed to the article and approved the submitted version.

## Funding

PF is supported by grant I01 BX004280, Biomedical Laboratory Research and Development (BLRD) VA MERIT Award Program, US Department of Veterans Affairs, United States of America.

## Conflict of Interest

The authors declare that the research was conducted in the absence of any commercial or financial relationships that could be construed as a potential conflict of interest.
